# Male Sex, Masculinization, Sexual Orientation, and Gynephilia Synergistically Predict Increased Sexual Jealousy

**DOI:** 10.1007/s10508-025-03225-z

**Published:** 2025-08-22

**Authors:** Leif Edward Ottesen Kennair, Mons Bendixen, David P. Schmitt

**Affiliations:** 1 Department of Psychology, Norwegian University of Science and Technology, 7491 Trondheim, Norway; 2 Department of Psychological Sciences, Kansas State University, Manhattan, KS USA

**Keywords:** Jealousy, Sex differences, Sexual orientation, Masculinization, Gynephilia

## Abstract

A robust, cross-cultural sex difference is typically found in jealousy responses to reveal emotional versus sexual infidelity. The only factor that seems to moderate this sex difference is sexual orientation, with male heterosexuals conspicuously reporting the greatest distress to sexual infidelity. Attempts at considering additional effects of psychological feminization and masculinization on jealousy responses have proven less reliable. In a sample of 4465 participants aged between 16 and 80 years (*M* = 35.41, *SD* = 13.07), including gay, lesbian, heterosexuals, bisexuals, and pansexuals, we examined how four indicators of feminization–masculinization were associated with sexual orientation and, subsequently, to jealousy responses to infidelity. We found that an exclusive gynephilic orientation and male sex in combination shifted the typical emotional jealousy focus toward more sexual jealousy. The four indicators of feminization–masculinization were linked to sexual orientation and, subsequently, to variable jealousy responses among men, but were only linked to sexual orientation among women. These findings support the position that female versus male mating psychology is not simplistically dimorphic. Instead, sex differences probably result from many different and continuous “psychological dials” that interact with each other, while also functioning in ways that are partially modular or independent.

**Supplementary Information:**

The online version contains supplementary material available at 10.1007/s10508-025-03225-z.

## Introduction

The experience of romantic jealousy can be profound, often with devastating consequences for individuals, their relationships, and society (Buss, 2013). From an evolutionary perspective, however, jealousy may have positive functions and serve, at times, as a component of an adaptive mate retention strategy (Buss, 1988; Buss et al., 1992; Daly et al., 1982). Indeed, jealousy is often activated by existential threats to one’s mateship, including suggestive cues to potential sexual infidelity by the mating partner, threats coming from would-be mate poachers, and any signals that one’s current mate might permanently defect from the relationship (Buss, 2013; Buss & Haselton, 2005). A proper functionalist perspective on jealousy requires the theoretical mapping of its psychological design, the inputs that activate it, and the outputs it is designed to produce (Cosmides & Tooby, 2015; Roney, 2016).

Buss et al. (1992) were the first to theoretically map out, and empirically confirm, sex differentiated functional design across different types of jealousy: When heterosexual men and women are confronted with infidelity dilemmas, a greater proportion of men, relative to women, report being more distressed by a partner’s sexual infidelity than by a partner’s emotional infidelity (Edlund & Sagarin, 2009). The evolutionary logic behind men’s relatively greater distress reaction to sexual infidelity is straightforward. Given internal female fertilization and gestation in humans, ancestral men (but not women) faced the adaptive problem of parental uncertainty. Moreover, ancestral men likely devoted considerable paternal investment in their offspring (Geary, 2000; Marlowe, 2000). This acute confluence of parental uncertainty combined with typically high paternal investment meant that ancestral human males who remained naïve about their partner’s extra-pair sexual behaviors with romantic rivals potentially suffered serious fitness costs (Buss et al., 1992).

Empirically, for over a quarter century, psychologists have consistently observed moderately-sized sex differences in the tendency to find sexual infidelity more distressing than emotional infidelity (Bendixen et al., 2015a; Buss, 2018; Edlund & Sagarin, 2017; Sagarin et al., 2012b). In that time, very few systematic moderators of this sex difference have been discovered (Bendixen & Kennair, 2023; Bendixen et al., 2015a; Brase et al., 2014; Larsen et al., 2021; Sagarin et al., 2012b). However, it is not the case that all men report sexual jealousy as more distressing than emotional jealousy, a common misinterpretation of the evolutionary perspective on jealousy (Buss & Haselton, 2005; Sagarin, 2005). Nor is it the case that women never experience sexual jealousy. Such binary, essentialist interpretations of evolutionary psychology are not uncommon, but are at odds with the foundational principles of evolutionary psychology (Schmitt, 2022). Rather, nearly all bona fide research investigations using separate measures of jealousy reactions to sexual and emotional infidelity report finding an overall interaction of sex and jealousy type—with men, relative to women, typically being more upset by sexual infidelity compared to emotional infidelity (Buss, 2018; Sagarin et al., 2012b).

The evolved adaptations that give rise to sexual and emotional jealousy are likely complex involving numerous genetic and neurological substrates (Kupfer et al., 2022), with somewhat different underlying psychological designs existing within men and women (Takahashi et al., 2006). Identifying these sex differences in psychological design and how their physical substrates reliably cause sexual jealousy to be experienced as more distressful than emotional jealousy in men (compared to women) has been a challenging task (Buss, 2018). Part of the psychological design in men that may yield a special intensity of sexual jealousy distress may include adaptations for men to selectively perceive and react to local costs of cuckoldry (Scelza, 2014; Zandbergen & Brown, 2015) with men expressing more distress to sexual infidelity in particularly high paternal investment cultures (Scelza et al., 2020).

Another potential intensifier of sexual jealousy responses might involve an interaction of sex and sexual orientation. Because cuckoldry risk is only present for men who are partnered with women, as opposed to men partnered with men, or women partnered with men or women (Sagarin et al., 2003), it may be that only men who are personally partnered with women experience intensified sexual jealousy relative to emotional jealousy (Frederick & Fales, 2016; Scherer et al., 2013; Valentova et al., 2020). We will consider this putative feature of the special design of sexual jealousy evocation in men—that sexual jealousy is particularly distressing for men when paired with a female partner.

Importantly, we examine here whether any heightened experiences of sexual jealousy in men (versus women) are linked in a dose-dependent manner with *dimensional* conceptions of sexual orientation (Lippa, 2020). We focus on rigorously evaluating whether sexual jealousy is particularly distressing among men with stronger gynephilic interests, and whether sexual jealousy is reduced among men with stronger androphilic interests. It is possible both are required, with heightened sexual jealousy only experienced when a specific combinatorial threshold of high gynephilia and low androphilia is crossed.

We also dimensionally examine whether heightened experiences of sexual jealousy co-occur with other widely documented and pervasive psychological differences between women and men, what have been termed dials of psychological gender (Schmitt, 2017). We evaluate whether sexual jealousy is particularly intensified among men with more masculine self-perceptions, more male-typical occupational interests, more systematizing (as opposed to empathizing) cognitive tendencies, or lower childhood gender non-conformity (Archer, 2019). Empirically situating heightened experiences of sexual jealousy within various indicators of dimensional sexual orientation and psychological gender—each with their own known genetic, neurological, endocrinological and developmental origins—could help to shed much light on the potential origins and special design features of sexual jealousy (Shirazi et al., 2020a, 2020b, 2022; Takahashi et al., 2006).

### Jealousy Activation and Sexual Orientation: On the Personal Costs of Cuckoldry

In contrast to consistent findings from studies that utilize only heterosexual samples (Sagarin et al., 2012b), sex differentiated patterns of jealousy are often not evident in samples of sexual minorities (Frederick & Fales, 2016; Harris, 2003; Sheets & Wolfe, 2001). In a pioneering evolutionary approach to jealousy in gays/lesbians, Symons (1979) suggested that gay men’s reduced sexual jealousy response might be the result of other aspects of male sexual psychology such as the general need for sexual variety suppressing experienced jealousy. An early test of the sex difference in jealousy that considered the effect of sexual orientation concluded that only heterosexual males were more distressed by the sexual aspect of infidelity (Sheets & Wolfe, 2001). Recently, two large studies reached similar a conclusion: no sex differences in experienced jealousy are reported for sexual minority groups (Frederick & Fales, 2016; Valentova et al., 2020). For people with less canalized sexual orientations (i.e., bisexuals), the reproductive threat-based evolutionary model of jealousy predicts that bisexual men with female long-term partners should be equally distressed by the sexual infidelity of their partner as heterosexual men are (Howard & Perilloux, 2017; Sagarin et al., 2012a; Scherer et al., 2013). Recently this is supported by the finding that heterosexual men did not differ from bisexual men when they responded about female partners when responding to forced-choice questions (Valentova et al., 2022).

### Jealousy and Psychological Gender: On the Origins of Sexual and Emotional Jealousy

Schmitt (2017) proposed that it may be especially useful to think of sex-specific adaptations—including jealousy-related mechanisms—as relatively independent psychological gender dials (rather than one dichotomous gender switch). Each psychological gender dial is expected to naturally develop and emerge over time, but to varying degrees and not in perfect concert with all other sex-specific adaptations within the same individual. This is, in part, because each gender dial may be specially designed to be calibrated by different features of the ontogenetic environment (e.g., prenatal, early childhood, or pubertal hormone exposures; Pasterski et al., 2015; Swift-Gallant et al., 2022), activated by only certain socioecological contexts (e.g., skewed operational sex ratios, high local pathogen levels, or high population densities; Davis & Arnocky, 2022; Sng et al., 2018), and evoked by only certain personal situational factors (e.g., high personal mate value, current relationship status, or good physical condition; Geary, 2016; Lukaszewski et al., 2014; Schmitt, 2022). As a result, evolved sex-specific psychological gender dials in humans are expected to be numerous, highly developmentally and situationally contingent, and only obliquely related to one another in producing behavior at the individual level. At the group level, in most cultures, male-specific gender dials should tend to be turned toward a more male-typical or masculine psychology as expressed within the average man compared to the average woman, and female-typical or feminine gender dials are likely to be expressed more within the average woman (Archer, 2019; Schmitt, 2022).

It is important to note that even if psychological gender dials such as male sexual jealousy are reliably influenced by genetic, hormonal, and neurological differences between women and men (Ngun et al., 2011; Ritchie et al., 2018; Ruigrok et al., 2014; Trabzuni et al., 2013), this in no way implies dimorphic and completely non-overlapping “female brains” and “male brains” exist in humans (Del Giudice et al., 2016; Phillips et al., 2019), with sexual jealousy always and only residing with a “male brain.” Gendered psychology in humans is almost always dimensional with a high degree of overlap between different groups of people, especially women and men (Hyde, 2005; Zell et al., 2015). Even the largest differences in women’s and men’s psychological traits tend to emerge in ways that are probabilistic, fluid, and heavily dependent on culture (Fausto-Sterling, 2012, 2019; Lippa, 2002; Walter et al., 2020).

### Sex- and Sexual Orientation Differentiation in Masculinization and Feminization

If heightened sexual jealousy is an evolved, adaptive sex differentiated response to the local and personal costs of cuckoldry, to what degree will other aspects of psychological masculinization versus feminization—in cognitive orientations, occupational preferences, play styles (Lippa, 2002)—contribute to the increased phenomenological experience of sexual jealousy? Determining which psychological gender dials co-occur with heightened sexual jealousy may reveal features of the jealousy mechanism’s genetic, hormonal, and neurological substrates, as well as its special design features. Previous attempts at considering masculinization and jealousy responses, such as linking it to digit ratios (2D:4D), have not provided conclusive answers (Bendixen et al., 2015b; Fussell et al., 2011).

Several underlying indicators of feminization and masculinization may be relevant for understanding the links between jealousy and sexual orientation. One much studied set of gender dials are systemizing versus empathizing cognitive orientations (Baron-Cohen, 2006; Baron-Cohen et al., 2005), where women or feminized individuals are higher on cognitive empathizing, while men or masculinized individuals are higher on cognitive systemizing. This is a difference that develops early in life and also has been associated with sexual orientation among men, with empathizing weaker and systemizing stronger among heterosexual versus gay men (Zheng & Zheng, 2015).

Further, occupational preferences are highly sex differentiated and show a clear Things versus People differentiation effect (Kuhn & Wolter, 2022). While one might expect such preferences to be less pronounced in more gender egalitarian societies, they are not, resulting in what is coined the Scandinavian paradox (Lippa, 2010). There is also an effect of sexual orientation on preferences for occupation, where gay men prefer jobs that typically are more dominated by women, and lesbian women to a larger degree than other women choose jobs that are dominated by men (Lippa, 2010; Tilcsik et al., 2015). Bisexual men and women’s preferences for male and female dominated jobs are typically intermediate of those reported by gays /lesbians and heterosexuals.

Childhood nonconformity to gender roles, including gendered play activities, are also clearly sex differentiated and predictive of sexual orientation (Bailey & Zucker, 1995; Bailey et al., 2016, 2020). Recently it was concluded that despite strong sociocultural attempts to influence the gendered pattern of play, there is no evidence of reduced sex differentiation across 50 years of research (Davis & Hines, 2021). Gay or lesbian individuals are less gender conforming across a range of behaviors, and analysis of childhood home videos show that this is observable at early age and consistent with self-report (Lippa, 2008a; Rieger et al., 2008).

Subjective gender role identity (i.e., feminine versus masculine) is also highly sex- and sexual orientation differentiated. Gay and bisexual men describe themselves as more feminine than heterosexual men, and lesbian and bisexual women are more masculine than heterosexual women (Lippa, 2008a; Rieger et al., 2008). Lippa (2008b) reported that bisexual men are more feminine than gay men across nations and regions, while bisexual women are less masculine than lesbians and more masculine than heterosexual women.

The above masculinization/feminization indicators may all reflect one underlying “psychological gender” construct or each might be an independent gender dial that contributes additively or interactively to an individual’s sex-typicality (Schmitt, 2017), including sex-typical patterns of jealousy. All of these masculinization/feminization indicators have shown to be somewhat sex- and sexual orientation differentiated, but the degree to which these factors are linked to sexual jealousy responses will not “explain away” sex differences in jealousy (i.e., “throwing the baby out with the bath water”, see Kennair et al., 2016; Schmitt et al., 2012). Instead, revealing links between psychological gender and sexual jealousy across sex and sexual orientation may help us understand the substrates through which sex differences in jealousy emerge. It may help us determine when, to what degree, and why different facets of sexual orientation intensify the expression of sexual jealousy.

### The Current Study: Aims and Hypotheses

We will examine the extent to which forced choice jealousy responses are linked to sexual identity of the respondent, current relationship status, the sex of the current romantic partner, and the basic level of attraction to same-sex or opposite-sex individuals. Further, we will consider how several indicators of feminization-masculinization including systemizing versus empathizing, childhood nonconformity to gender roles (gendered play activities), gender role identity (i.e., femininity and masculinity), and vocational preferences predict sexual orientation and jealousy responses.

We will be testing the following hypotheses:

H1: We expect to find a robust sex difference in jealousy responses between heterosexual men and women, typical of previous Scandinavian studies Bendixen et al (2015b).

H2: We expect to replicate the finding of no sex differences in jealousy responses among the sexual minority groups (i.e., gays versus lesbians and bisexual females versus bisexual males) who are all expected to be dominated by emotional jealousy (Fredrick & Fales, 2016).

H3: In line with Sagarin et al. (2012a) reproductive threat-based model, jealousy responses of bisexual males ought to be similar to heterosexual males if their current partner’s sex is female. However empirical findings suggest that sexual orientation reduces the sex difference (Frederick & Fales, 2016; however, see Howard & Perilloux, 2017).

H4: For jealousy responses we expect to find similar effects for a continuous measure of gynephilic-androphilic preference as for self-identified sexual orientation (i.e., the sex difference will be reduced for those who do not report a strict heterosexual orientation).

H5: Within each sex we expect—using structural equation models (SEM)—that the feminization-masculinization construct (the four interlocking dials; gendered child play, systematizing/empathizing, gender role identity, and vocational preferences) will predict sexual orientation (gynephilic versus androphilic preference) *and* jealousy responses (Schmitt, 2017). Specifically, we expect higher masculinization to be associated with more gynephilic preference and with being more upset by the sexual aspect of the infidelity for both sexes. In addition, we will examine whether jealousy responses are affected by the feminization-masculinization construct directly or if they are mediated by sexual orientation (gynephilic versus androphilic preference).

## Method

### Participants

A cross-sectional study covering one sample of the general Norwegian population (*n* = 3738), and one sample of Norwegian sexual minorities (*n* = 807) was carried out. We invited participants between ages 16 and older to respond to a web-based questionnaire covering reactions to imagined infidelity, various indicators of psychological masculinization and femininization, sexual orientation and erotic preferences, sociosexuality and demographics.

Data were subject to cleaning procedures for identifying extreme or monotonous responding and resulted in the removal of 9 respondents. Available for analyses were 3296 who self-identified as heterosexual, 386 bisexual, 646 gay/lesbian, 137 pansexual, and 71 as transgender. For the scope of this paper, those with transgender identity were excluded, leaving *N* = 4465 eligible for analysis. Each participant identified their sex (man or woman) based on the sex they were “assigned at birth” (optional with comments). A separate question on subjective sexual identity was posed thereafter, allowing for non-binary responses and an open field for comments. Participants were aged between 16 and 80 years old (*M* = 35.41, *SD* = 13.07). The majority reported being in a romantic relationship (men: 60%, women: 68%).

### Procedure

For the general sample, the study was announced on a Norwegian project page on Facebook during the fall of 2016 using paid advertisement. This procedure acquired responses from a minimum number of participants for predefined age and gender strata. By clicking on advertisement link, the participants were redirected to the web survey. For the minority sample, the study was announced on a national LGBTQ homepage, and on the LGBTQ youth organization homepage providing a link to the web survey during the spring of 2016. When activating the link to the web survey, each participant received written information on the purpose of the research, what questions would be posed, possible discomfort related to responding, and their rights to withdraw from the project. They were further informed that participation was voluntary. Because of the sensitive nature of several of the questions regarding sexuality, sexual identity and sexual orientation, each participant was strongly encouraged to respond to the questionnaire in private.

### Measures

#### Jealousy Responses to Infidelity

We applied the four most frequently used forced choice scenarios from Buss et al. (1999) infidelity dilemmas questionnaire. The items have previously been translated to Norwegian and applied by Bendixen et al. (2015a).

The scenarios covered the following dilemmas: (SC1) Enjoying a sexual relationship versus forming deep emotional relationship, (SC2) Trying different sexual position versus falling in love, (SC3) Given both an emotional attachment and sexual intercourse, which aspect of the infidelity would upset you more, and (SC4) Emotional attachment but no sexual intercourse versus sexual intercourse but no emotional attachment. For each dilemma, the participants selected the aspect that was more upsetting (emotional or sexual). Responses to the four forced choice scenarios were coded 0 (*the emotional infidelity more upsetting*) and 1 (the *sexual infidelity more upsetting*). Internal consistency was good (total: KR-20 = 0.80, men: KR-20 = 0.81, women: KR-20 = 0.76). The items scores were averaged to form a Jealousy Scale. The scores reflect the percentage of scenarios (from 0 to 100%) in which the sexual aspect is reportedly more upsetting than the emotional aspect of the imagined infidelity. Higher scores are associated with greater discomfort with sexual infidelity relative to emotional infidelity.

#### Gynephilic-Androphilic Sexual Attraction

How strongly the participants were sexually attracted to men (Androphilic) and women (Gynephilic), respectively, was measured with two questions from the BBC Internet Survey (Lippa, 2008b). A 7-point response scale was used with endpoints 1 (*not at all*) and 7 (*very much*). Overall, sexual attraction toward men was negatively associated with sexual attraction toward women (*r* =  − 0.82), but more strongly so for male (*r* =  − 0.89) than for female participants (*r* =  − 0.55). For analysis, we computed a mean score of attraction toward women and reversed attraction toward men. To ease interpretation, we subtracted 4 from the scale so that positive scores reflect degree of gynephilic sexual attraction, negative scores reflect degree of androphilic attraction.

### Indicators (Dials) of Feminization-Masculinization

#### Empathizing and Systemizing

The participants’ self-reported empathic capability and ability to analyze and construct systems was measured with the Brief EQ and SQ Questionnaires (Manning et al., 2010). Sample items for empathizing were “I really enjoy caring for other people” and “I usually stay emotionally detached when watching a film” (reversed). Sample items for systemizing were “I find it easy to grasp exactly how odd work in betting” and “I can easily visualize how the motorways in my region link up.” The participants rated their responses on a 4-point Likert scale. Item scoring and of the scaling of EQ and SQ closely followed the instructions given by Manning et al. (2010). Internal consistencies (Cronbach’s alpha) were 0.64 and 0.69 for EQ and SQ, respectively, and slightly lower when analyzed separately for men and women. For analysis, we computed difference scores S-E (subtracting EQ from SQ), with positive scores reflecting systemizing over empathizing.

#### Gender Non-Conformity

Lack of conformity to sex-typed behavior in childhood years (age 12 and below) was measured with the Childhood Gender Nonconformity Scale (Lippa, 2008a; Rieger et al., 2008). There were 7 statements for each sex. Sample items for men were “I was a feminine boy” and “I preferred playing with girls rather than boys.” Sample items for women were “As a child I was called a ‘tomboy’ by my peers” and “As a child I preferred playing with boys rather than girls.” Participants rated their agreement with each statement on a 7-point Likert scale ranging from 1 (*strongly disagree*) to 7 (*strongly agree*). Internal consistency (Cronbach’s alpha) was good (men: 0.82, women: 0.82). For each sex we averaged the item scores. Higher scores reflect more nonconformity to sex-typical play. However, for the SEM analysis, item scores for men were reversed (i.e., higher scores = stronger preference for masculine play during childhood for both sexes).

#### Male-Typical versus Female-Typical Occupation Preferences

We applied the 10-item BBC Internet Survey measure of gender-related occupational preferences (Lippa, 2008b). The participants rated—regardless of their current occupation or occupation status—their preferences for each type of occupation on a 7-point Likert scale with anchors 1 (*strongly dislike*) and 7 (*strongly like*), and mid-point 4 (*neither like nor dislike*). Internal consistencies (Cronbach’s alphas) for ipsatized items were 0.75, 0.74, and 0.74, for all participants, men and women, respectively. In line with Lippa (2008b), we computed a scale reflecting occupational preferences with higher scores reflecting stronger preference for male-dominated jobs and lower scores reflecting stronger preferences for female-dominated jobs.

#### Perceived Masculinity-Femininity

Based on the work by Lippa (2008a) we posed two questions for measuring degree of self-perceived masculinity and femininity: (1) “Relative to others of our sex and age, how masculine or feminine do you *feel*?” and (2) “Relative to others of our sex and age, how masculine or feminine do you think *others perceive you*?” The participants responded to a 7-point Likert scale with anchors 1 (*very masculine*) and 7 (*very feminine*), and mid-point 4 (*equally masculine and feminine*). The two items overlapped considerably (total: *r* = 0.89, men: *r* = 0.82, women: *r* = 0.78). For analysis, the item scores were reversed (high scores reflect more masculinity) and averaged.

These four indicators were moderately associated, mean *r* = 0.32 [0.22–0.47] for women and mean *r* = 0.31 [0.15–0.40] for men, as would be expected if evolved sex-specific psychological gender dials in humans are numerous, highly developmentally and situationally contingent, and only obliquely related to one another at the individual behavioral level.

### Analyses

For testing sex, sexual orientation, and relationship-status group differences, we applied ANCOVAs or one-way analysis of variance, accounting for any possible effects of age. Further, OLS regressions were applied for examining linear and curvilinear effects of Gynephilic versus Androphilic attraction in men and women with robust estimation of standard errors. Path analyses were performed using latent structural equation model (SEM) on samples of men and women separately. We applied the maximum likelihood with missing values option that imputes missing values. This retains maximum number of cases. Goodness of fit indexes are reported for each model (Hu & Bentler, 1999). Indirect (mediation) effects were examined using the MEDSEM package in Stata (Mehmetoglu, 2017) that applies Zhao et al.’s (2010) approach to mediation and performs a Monte Carlo test with 5000 replications.

For simple sex differences we report on Cohen’s *d* (with unequal variances) and common language effect size (CL, McGraw & Wong, 1992). All statistical tests were performed using StataNow/MP 18.5 for Mac (StataCorp., 2023).

## Results

### Descriptive Statistics

An overview of heterosexual, bisexual, gay/lesbian, and pansexual men and women’s age, relationship status, and their average scores on the four indicators of masculinization and sexual preference is found as supplementary material. Heterosexuals were slightly older than non-heterosexuals, and they were also more often currently partnered. Relative to all other groups, heterosexual men scored higher on systemizing-empathizing, higher on masculine childhood play, higher on male occupation preferences and subjective feelings of being male. Heterosexual men and lesbian women evinced the strongest attraction to women (i.e., strong gynephilic orientation). Relative to the other groups, gays and lesbians reported the highest levels of nonconformity to sex-typed behavior.

Regarding perceived feelings of being masculine or feminine, heterosexual more than bisexual men reported being masculine, and bisexual men more than gay and pansexual men (for details, see Supplementary Material. Heterosexual women reported being more feminine than bisexual and pansexual women, while lesbians on average reported being least feminine. Bi-and pansexuals reported markedly less canalized gynephilic-androphilic preferences (i.e., closer to zero) compared to heterosexuals and gay/lesbians.

### Jealousy Responses in Heterosexuals, Bisexuals, and Gay Men and Lesbian Women

To test Hypothesis 1, that heterosexual men, relative to heterosexual women, would report more jealousy imagining their partner’s sexual rather than emotional infidelity, we first performed a simple *t*-test comparing men’s and women’s Jealousy Scale scores, with higher scores indicating sexual infidelity is more upsetting than emotional infidelity. Among heterosexuals, men scored markedly higher on the Jealousy Scale than women, *t*(3283) = 18.16, *p* < 0.001, *d* = 0.63 [0.56, 0.70] / CL = 0.67. As seen in Table 1, 58.5% of heterosexual men were more or equally upset by the sexual aspect of the infidelity relative to the emotional aspect when the scores across the four scenarios were averaged, compared to 30.7% of heterosexual women, *χ*^2^(1, *N* = 3285) = 256.75, *p* < 0.001.

**Table 1:** Mean Jealousy Scale score, 95% CI’s and proportion being equally or more upset by the sexual aspect) for men and women across sexual orientation

	Men	% > .50	Women	% > .50
Heterosexual	0.50 [0.48, 0.52]	58.5	0.27 [0.25, 0.29]	30.7
Bisexual	0.33 [0.26, 0.40]	40.0	0.25 [0.21, 0.30]	29.0
Gay/Lesbian	0.25 [0.22, 0.29]	30.0	0.32 [0.27, 0.37]	34.7

To test Hypothesis 2, we conducted a 2 (sex: man versus woman) × 3 (sexual orientation: hetero versus bi versus gay/lesbian) analysis of covariance (ANCOVA), controlling for the effect of age. We included age as a covariate because age differed across the sexual orientation groups. Interactions were examined and reported when significant. Because of low number of pansexual men (*n* = 33), we excluded pansexuals from this analysis.

**Fig. 1:**
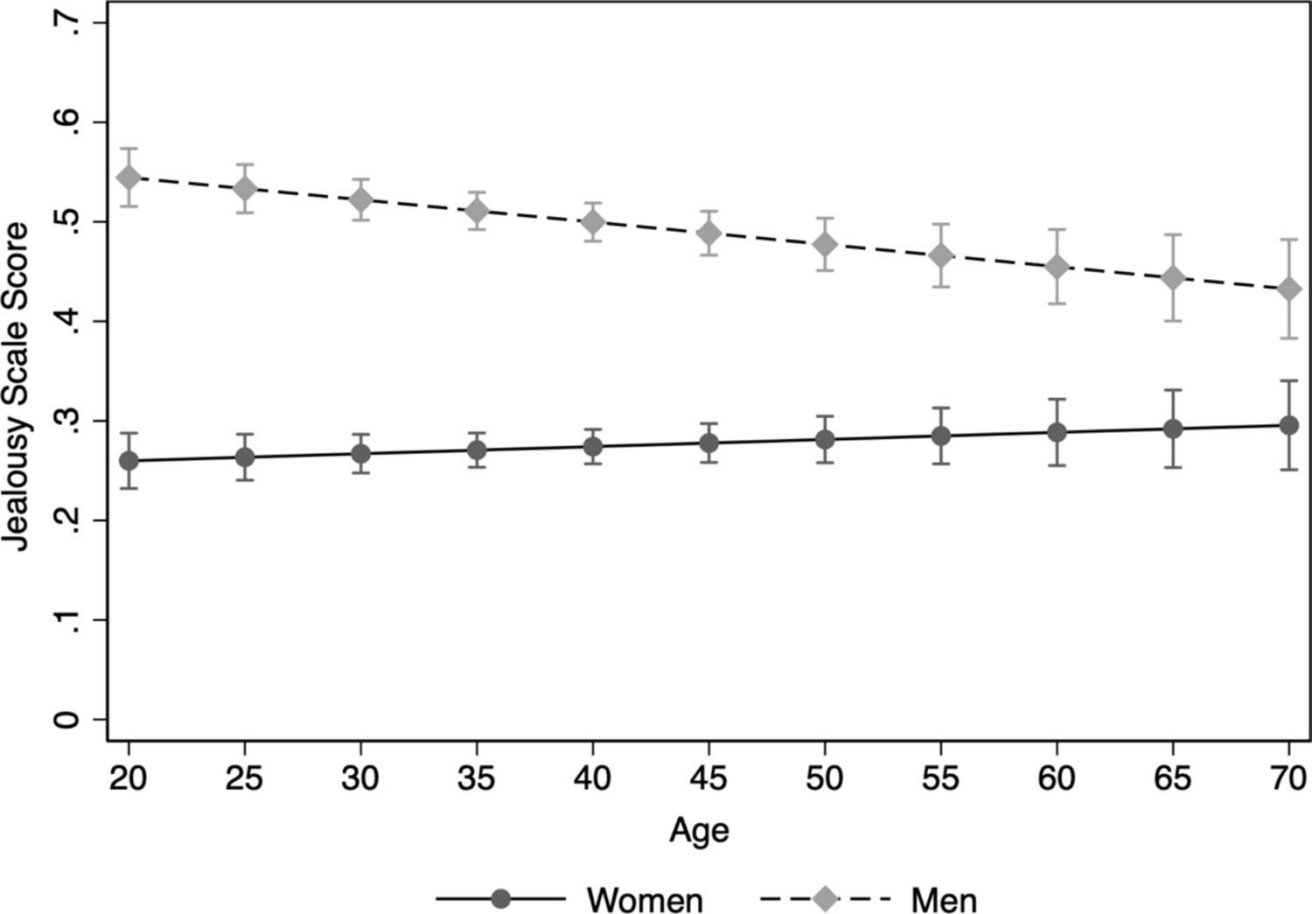
Marginal means and 95% CIs for Sexual Jealousy scores for men and women across the life span

The Jealousy Scale responses differed for men and women, *F*(1, 4305) = 27.10, *p* < 0.001, and across the three sexual orientation groups: *F*(2, 4305) = 25.71, *p* < 0.001. A significant sex x sexual orientation effect, *F*(2, 4305) = 44.99, *p* < 0.001, suggests that the sex effect differed across sexual orientation groups. Bisexual men scored slightly higher than bisexual women (*d* = 0.24 [–0.02, 0.47] / CL = 0.57). For gays/lesbians, the sex difference was reversed with women scoring slightly higher than men (*d* =  − 0.17 [–0.34, 0.01]/ CL = 0.45). The 95% CIs of these sex differences suggest they were not different from zero. There was no main effect of the covariate age (*F* = 1.08, *p* = 0.31), but the sex effect was moderated by age, *F*(1, 4305) = 9.46, *p* = 0.002. Across the life span men and women’s jealousy scores became more similar (see Fig. 1). However, this sex x age interaction effect was present for heterosexuals only, *F*(1, 3286) = 9.14, *p* = 0.003.

Given the sex x sexual orientation interaction effect above, we examined which sexual orientation groups differed within each sex. We performed one-way analysis of variance with post-hoc tests (Bonferroni correction) for each sex. For men, there was an effect of sexual orientation, *F*(2, 2081) = 74.15, *p* < 0.001. The post-hoc tests showed that heterosexuals scored significantly and moderately higher on the Sexual Jealousy scale than both bisexuals (*d* = 0.43) and gays/lesbians (*d* = 0.64). Bisexuals and gays/lesbians did not differ significantly from one another (*p* = 0.200, *d* = 0.22). For women, the effect of sexual orientation did not reach significance (*F*(2, 2226) = 2.60, *p* = 0.075), and the post-hoc tests did not reveal any significant differences between any of the three sexual orientation groups.

### Heterosexual and Bisexual Men and Women with Opposite Sex Partners

**Fig. 2:**
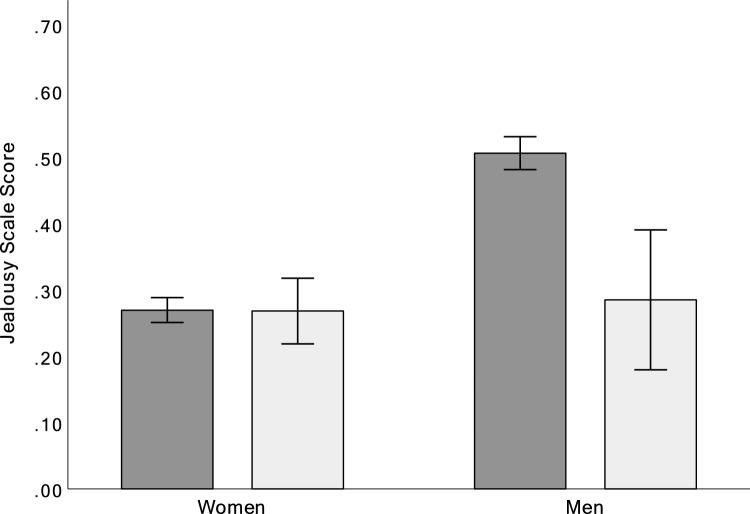
Jealousy score for heterosexual (dark gray) and bisexual (white) women and men with opposite-sex partners

To test Hypothesis 3 that jealousy responses of bisexual males ought to be more similar to heterosexual males if partner’s sex is more important than an individual’s sexual orientation, we compared bisexuals living with an opposite-sex partner (31 men and 127 women) with heterosexuals living with an opposite-sex partner (819 men and 1088 women) in a 2 (sex) × 2 (sexual orientation) ANCOVA, controlling for the effect of age. The sex x sexual orientation interaction was significant,* F*(1, 2055) = 8.05, *p* = 0.005. Post-hoc comparisons showed that heterosexual men were moderately more jealous of the sexual aspect of the infidelity than bisexual men living with a woman (*d* = 0.54). Comparably, heterosexual women did not differ from bisexual women living with a man (*d* = 0.03). As shown in Fig. 2, it was only heterosexual men living with an opposite-sex partner who reported being more upset by imagining their partner’s sexual infidelity than their emotional infidelity.

### Gynephilic–Androphilic Preference and Jealousy Response

**Fig. 3:**
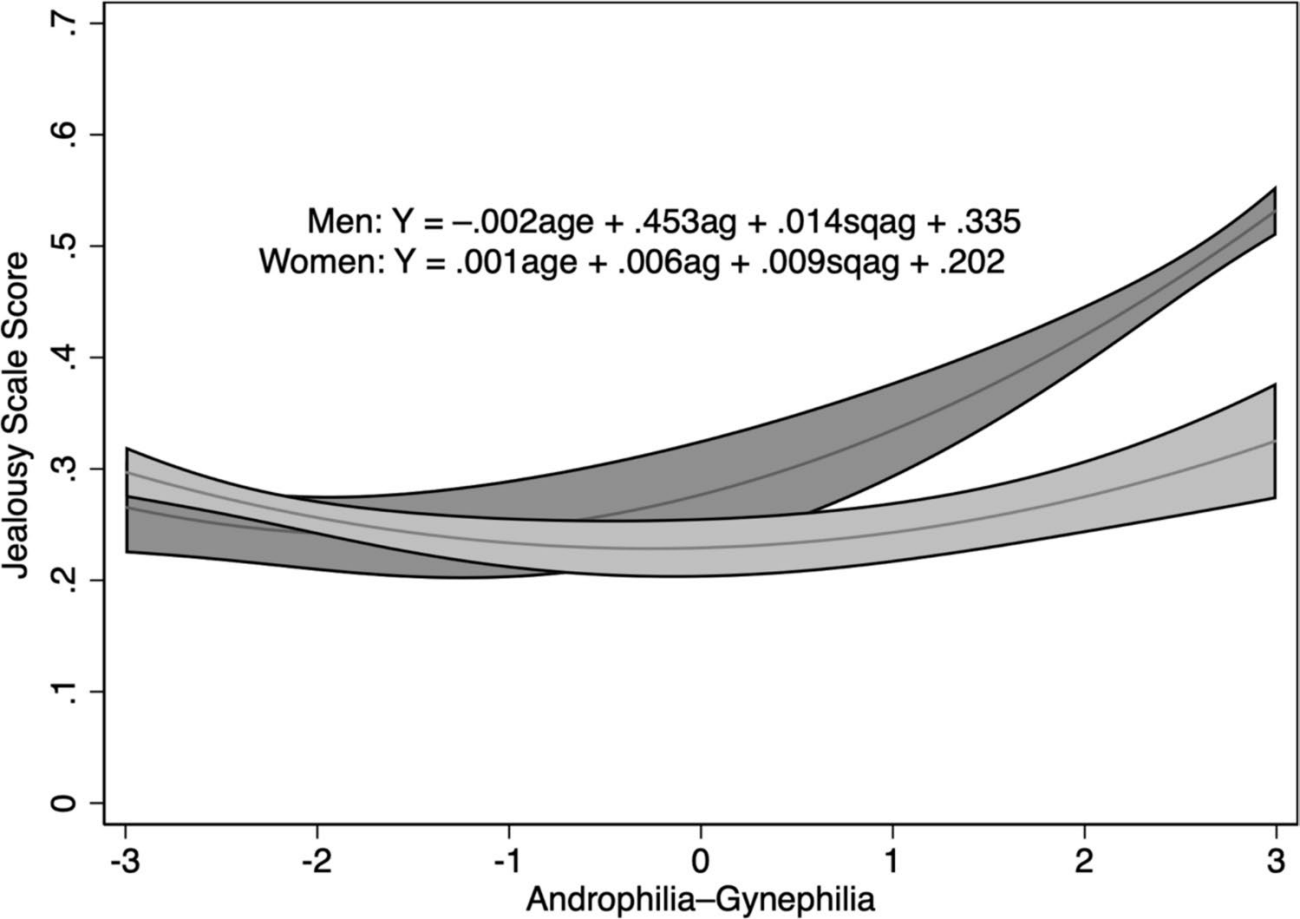
Mean and 95% CIs for jealousy scores as a function of level of androphilia (negative scores) versus gynephilia (positive scores) for men (dark gray) and women (light gray)

Moving beyond categories of sexual orientation, we measured strength of erotic attraction to men (androphilia) and to women (gynephilia) to permit a more fine-grained analysis of how erotic attraction to men and women is related to heightened sexual jealousy responses. To test Hypothesis 4, we regressed jealousy responses on age and gynephilic-androphilic erotic orientation in men and women separately. In addition, we examined potential curvilinear associations between erotic orientation and jealousy response by adding a quadratic gynephilic-androphilic term to the model. For men, this model accounted for a significant proportion of the variance in jealousy responses, *F*(3, 2094) = 62.34, *p* < 0.001, *R*^2^_adj_ = 0.081. Age had a significant effect, *t*(2094) =  − 2.87, *p* = 0.004, *b* =  − 0.060, and erotic orientation was both linearly (*t*(2094) = 11.91, *p* < 0.001, *b* = 0.255) and curvilinearly (*t*(2094) = 4.15, *p* < 0.001, *b* = 0.089) associated with jealousy responses. As shown in Fig. 3, men reported markedly higher levels of sexual jealousy only at high levels of gynephilia.

For women, the model accounted for less than 1% of the variance in jealousy responses (*F*(3, 2282) = 5.40, *p* = 0.001, *R*^2^_adj_ = 0.006). Neither age (*t*(2282) = 1.57, *p* = 0.117) nor erotic attraction (*t*(2282) = 1.32, *p* = 0.187) had any effect on jealousy responses, but erotic attraction did show a curvilinear association (*t*(2282) = 3.62, *p* < 0.001, *b* = 0.082). As shown in Fig. 3, women with either a stronger androphilic or a gynephilic erotic orientation tended to report being more upset by the sexual aspect of the infidelity relative to women reporting no clear preference. However, the gynephilia effect was not as strong as that for men.

### Feminization-Masculinization as Predictor of Gynephilic–Androphilic Preference and Jealousy Response in Men and Women

We first tested Hypothesis 5 applying a latent structural equation model (SEM) on the male sample predicting jealousy response (four scenarios) from the Masculinity-Femininity (M-F) construct (four indicators), accounting for the effect of age. Scoring higher masculinity on the M-F construct was clearly associated with being more upset by the sexual aspect of the infidelity (*b* = 0.215, *z* = 7.18, *p* < 0.001). Age was associated with both jealousy response (*b* =  − 0.088, *z* =  − 3.38, *p* < 0.001) and with the M-F construct (*b* = 0.270, *z* = 9.94, *p* < 0.001) suggesting that with increasing age men reported to be less upset by the sexual aspect of the infidelity and to be more masculine.

Next, we predicted Gynephile-Androphile (G-A) sexual attraction from Masculinity-Femininity (M-F) construct accounting for the effect of age. M-F showed a very strong and positive association with gynephile attraction (*b* = 0.696, *z* = 31.77, *p* < 0.001). Age was slightly negatively associated with gynephile attraction (*b* =  − 0.089, *z* =  − 4.30, *p* < 0.001).

**Fig. 4:**
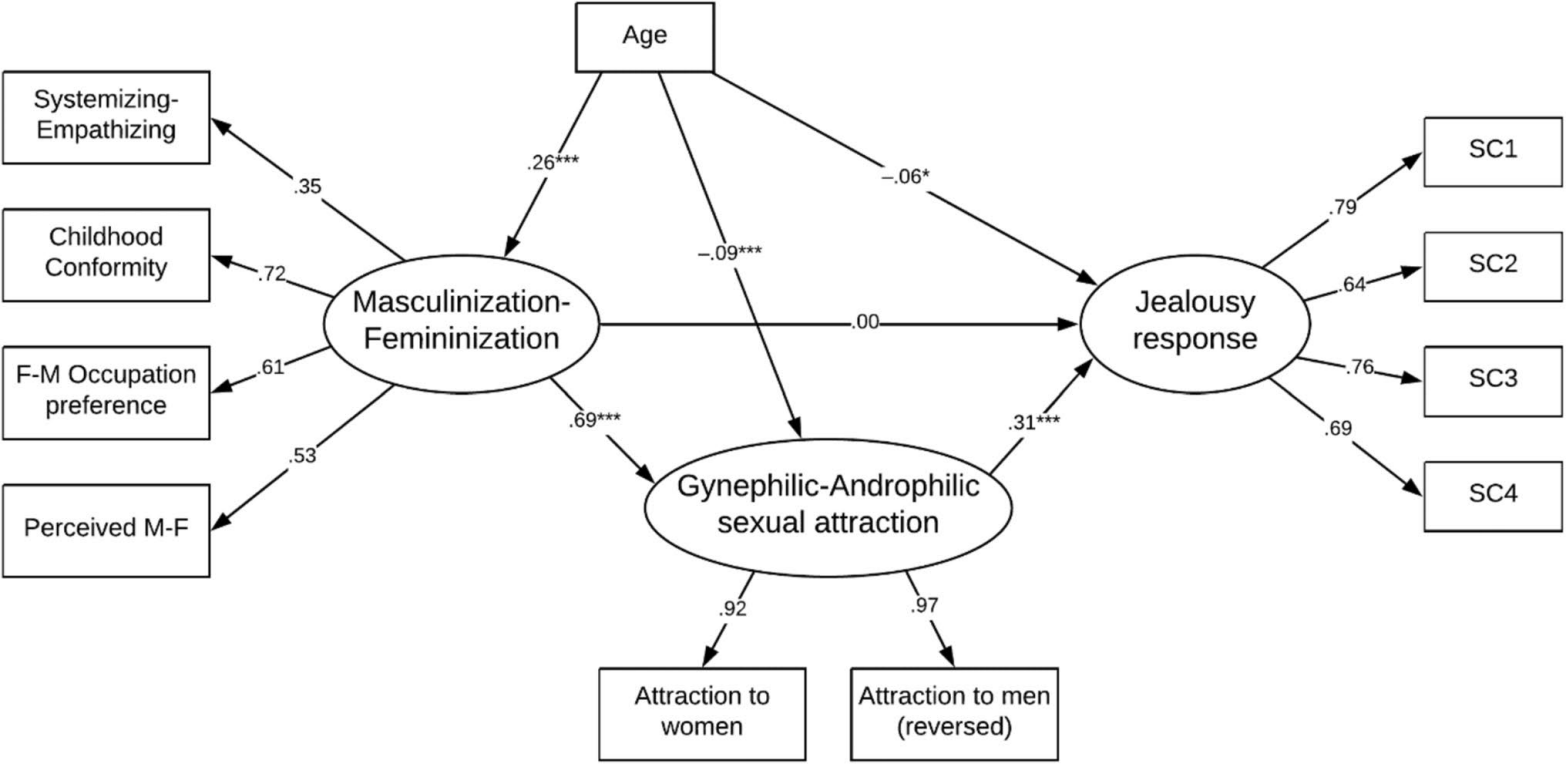
Predictors of jealousy responses for men. *p < .05, **p < .01, ***p < .001. Goodness of fit indexes with missing values imputed: *χ*^2^(39) = 383.05, *p* < .001, RMSEA = 0.064 [0.059, 0.070], CFI = 0.958, TLI = 0.941

Finally, we tested a compete model as outlined in Fig. 4 with the M-F construct as the primary predictor of jealousy response and G-A attraction as mediator. We accounted for the effect of age on the other latent variables in the model. Once the effect of G-A attraction was accounted for the M-F construct no longer predicted Jealousy. Gynephilic attraction was moderately associated with more sexual jealousy. From the above regression analyses of curvilinear effects, the underlying association is likely to be even stronger. We applied the Zhao et al. (2010) approach to testing mediation effect. This performs a Monte Carlo test with 5000 replications (MEDSEM; Mehmetoglu, 2017). The indirect effect of M-F on jealousy response was significant (*b* = 0.215, *z* = 8.32, *p* < 0.001) but the effect was fully mediated by the G-A attraction construct. Applying Hu and Bentler’s (1999) cutoff criteria for multiple indices the data provided a good fit for the model.

**Fig. 5:**
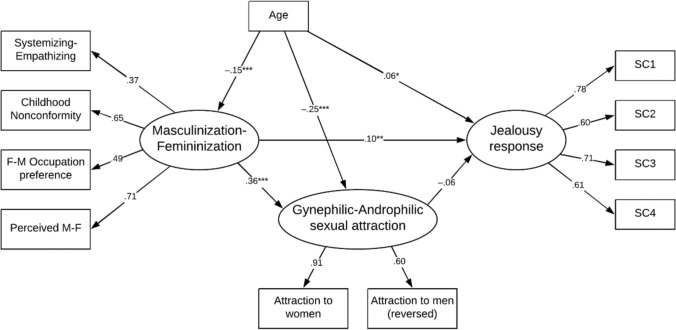
Predictors of jealousy responses for women. *p < .05, **p < .01, ***p < .001. Goodness of fit indexes with missing values imputed: *χ*^2^(39) = 432.36, *p* < .001, RMSEA = 0.066 [0.060, 0.071], CFI = 0.921, TLI = 0.889

We repeated the above three SEM analyses for women. In the first model, and when the effect of age was accounted for, higher masculinity scores on the M-F construct was associated with more sexual jealousy (*b* = 0.076, *z* = 2.56, *p* = 0.010). A direct comparison of the beta’s for men and women (Paternoster et al., 1998) showed that the effect was significantly smaller for women (*z* = 4.16, *p* < 0.001). In the second model, masculinity was moderately associated with G-A attraction (*b* = 0.362, *z* = 7.03, *p* < 0.001). Again, this effect was significantly smaller that the corresponding effect for men (*z* = 8.86, *p* < 0.001). Finally, and as shown in Fig. 5, when we added G-A attraction to the first model, there was no change in the effect of the M-F construct on jealousy response (hence, no mediation). The effect of G-A attraction on jealousy response was not significant in this model (*b* =  − 0.061, *z* =  − 1.88, *p* = 0.060), but the effect is likely to be curvilinear (as shown in Fig. 3 above). The data provided an adequate fit for the model (Hu & Bentler, 1999).

## Discussion

The sex difference in the relative import of emotional versus sexual jealousy has to a large degree been presented as a difference due to evolved sexuality. During the last two decades a growing number of studies have tested moderators and mediators of experienced jealousy, without finding any large impacts on the observed sex difference (Bendixen & Kennair, 2023; Bendixen et al., 2015a, 2015b; Brase et al., 2014; Larsen et al., 2021; Sagarin et al., 2012a, 2012b). However, one factor does seem to moderate this sex difference: categorical sexual orientation (Frederick & Fales, 2016). Is this moderation effect also evident with a more continuous androphilic-gynephilic approach to sexual orientation? And are sexual orientation and jealousy responses both predicted by a similar set of established feminization-masculinization dimensions? The current paper set out to address these questions in greater detail than previous research has, with the goal of elucidating the functional design and causal origins of sex differences in sexual jealousy.

We found, as hypothesized (H1), the typical marked, sex difference in jealousy responses among Scandinavian heterosexual participants, with heterosexual men expressing more pronounced upset at sexual compared to emotional infidelity. This sex difference was partly moderated by age, suggesting that jealousy responses are less sex differentiated at older ages. Also, reproducing the findings from most investigations of the effect of sexual orientation, we found support for Hypothesis 2 that the sex differences within self-reported categorical groups of sexual minorities were marginal or reversed. Being male is not enough, as only heterosexual males express greater upset at sexual compared to emotional infidelity. These findings were further replicated when considering the effects of continuous measures of sexual orientation (Hypothesis 4): distress primarily of the sexual aspect of infidelity was driven by exclusive gynephilia in addition to male sex. Any amount of androphilia, and female sex, act as functional breaks on the sexual jealousy mechanism, switching the focus toward relational or emotional aspects rather than sexual infidelity. Also, the effects of gynephilia and androphilia were less apparent in women.

The finding that there was no sex difference among bisexual men dating women and bisexual women dating men is a challenge for an evolutionary approach and was from a reproductive-threat perspective an unexpected finding (Sagarin et al., 2012a, 2012b). From this perspective, bisexual men dating women should be equally distressed by the sexual infidelity of their partner as heterosexual men are (Hypothesis 3). Throughout evolutionary history, men who mated with women and who invested in offspring they assumed to be their own were in danger of cuckoldry, independent of any core sexual orientation or proclivity toward androphilia. We do not know whether this may be related to less paternal investment or whether there is more extra-pair behavior among bisexual fathers, although these factors might explain the current findings.

Finally, in support of Hypothesis 5, we found that four indicators of femininization-masculinization influence both androphilia-gynephilia dimensions and jealousy responses for men, while these sex differential dials only predicted sexual orientation in women, and to a lesser degree. This is likely, in part, because there is little differentiation of jealousy responses across sexual orientation in women. The effect of femininization-masculinization on jealousy was fully mediated by androphilia-gynephilia construct in men. As there is no effect of sexual orientation on jealousy response within women, there was no such mediation effect for women.

The current feminization-masculinization construct employed in this study has identified several interrelated dials. Preferences for gender non-conforming play are strongly related to the construct of femininization-masculinization for both sexes. Thus, this construct is probably based on precursors for later psychosexual development. For women, perceived, subjective masculinity-femininity (gender role identity) was similarly highly related to this construct. For men occupational preferences also was highly related to the feminization-masculinization construct. The weakest related dial for both sexes was the empathizing-systemizing variable, a dial that largely can differentiate between the sexes, but seems to be less related to our general construct of feminization-masculinization. In future studies, one might not need to keep the empathizing-systemizing scales as they also were the least reliable measures.

Different from prior studies that looked at sex and sexual orientation differences in these indicators separately, this study examined how strongly they were interrelated and how they in concert add to an underlying construct that predicts both gynephilic versus androphilic attraction and jealousy response in men and women. Future research may elaborate on this by including even further dials. Nevertheless, the current study has, without addressing any items directly related to sexual attraction, shown a high ability to predict continuous androphilic-gynephilic attraction in both men and women.

The idea that when a phenomenon is sex-linked no other feminization-masculinization factor may influence the phenomenon apart from biological sex, is probably widespread (e.g., Sheets & Wolfe, 2001). This is often combined with simplified notions of what innate or evolved actually means, and how to understand developmental processes underlying individual differences in sexual psychology. The integrated study of an evolved adaptation demands considering several different aspects including both past selection forces and current environmental inputs, including ontogenetic, socioecological, and personal situation factors (Schmitt, 2022).

Focusing on relational/emotional jealousy seems to be the normal state in most humans (i.e., stronger jealousy responses to emotional infidelity than to sexual infidelity). Deviation from this pattern is predicted to reflect degree of masculinization and may be associated with both sex and sexual preference. One may always discover or learn the negative effects of losing friendships, affiliates, and investment ontogenetically. These effects may be observed in others, or one may experience them oneself. There is a large literature on the affiliative adaptations of humans (Cosmides & Tooby, 2015), rarely does this literature focus on sex-specific processing. Further, such processes will be based upon an evolved social and relational psychology. However, while there are ontogenetic explanations of affiliative and relational jealousy, the negative emotional and fitness effects of cuckoldry may only have been established through selection. There is no feedback aversive ontogenetic mechanism that may have given us the individual psychological response, or the cultural practices involved. Even Skinner (1981) accepted that the only explanation given no feedback mechanism, would have to be selection.

How do the masculinization and feminization dials result in this pattern of findings? We have measured developmentally relevant aspects of identity, preferences, and abilities or traits. None of these are *sexual* in their content, and yet they present as more or less coordinated dials that in concert predict sexual orientation for both sexes. The systematic association between the dials is clearer for men, probably due to men’s sexuality being more canalized (Howard & Perilloux, 2017). However, the prediction of female sexual orientation is still moderately strong. And for men, as there is systematic variance in men’s responses tied to orientation, we therefore also see how these dials predict sexual jealousy responses. The findings support the general approach of dials, and the need to consider a continuous conceptualization of sexual orientation. We would recommend this psychological conceptualization of sexual orientation over the broader and coarser socially constructed categories of heterosexual, bisexual, and gay/lesbian.

There are two major theoretical questions that will demand further theoretical and empirical investigation. First, why are bisexuals with female partners not equally sexually jealous as heterosexuals? Second, why is the sexual jealousy response so sensitive? Even the slightest androphilic tendency/drop of gynephilic orientation seems to predict a lowering of the sexual jealousy aspect. The evolutionary puzzle is that throughout history probably few men who were exclusively gay became our modern-day ancestors. And yet, any men—gay, straight, or bisexual—who invested in their assumed own offspring would have benefitted evolutionarily from avoiding cuckoldry. Also, given that the mechanism of heighted sexual jealousy is present in most heterosexual men, the expectation would rather be that it would be selected for even when androphilia is present and be only primarily tied to gynephilia or to male sex. This certainly is not the empirical case, given the available data, and rather robust findings given sample sizes and effects. What this may tell us about the evolutionary, developmental, endocrinological, and psychological processes involved in male sexual jealousy specifically, and sex differentiated mechanisms in general, remains to be discovered. We hope this puzzle will be addressed in the future by cross-disciplinary researchers interested in these processes.

### Limitations and Future Research

Despite a large number of participants with sexual minority identity and detailed investigation of the different sex differentiation dials, the study has a few noteworthy limitations: First, the study design, with the use of online ads and convenience sampling both represent limitations to the representativeness of the sample, however, this was the only way to secure a large enough non-heterosexual sample size. Second, we also utilize some retrospective recall of childhood sex-typed behavior, which may introduce recall bias. Third, the use of hypothetical scenarios as measures of jealousy reactions may differ from reactions to real infidelity (Edlund et al., 2006). Finally, the tests of heterosexual and bisexual women and men with opposite-sex partners covered particularly few bisexual men with female partners, which adds some uncertainty to the strength of the effect size. While this does not suggest power issues in general, this specific issue nevertheless warrants future attention. Such research might also consider whether bisexual fathers invest less in their current relationship and in their children, which hypothetically might address this unexpected finding.

Further work needs to be done on why lack of androphilic orientation is vital to sexual jealousy coming online. Also, sexual versus relational jealousy, despite the forced choice paradigm, is not a question of either/or, but a question of degree given the variance across several scenarios. It is therefore unclear why all sub-samples—apart from exclusively gynephilic males—are so similar on the sexual jealousy dimension, reporting in average a low but present elevation across groups. There is therefore greater detail to be mined in the understanding of emotional versus sexual jealousy, even within subgroups that generally are most distressed by the emotional aspects of jealousy.

### Conclusions

The robust sex difference of sexual versus emotional jealousy in heterosexual samples is not merely a simple sex difference in psychology. It is also a function of continuous, dimensional features of sexual orientation. Strong gynephilic attraction and low levels of androphilic orientation, when combined with male sex, powerfully shift the general relational and emotional focus of male individuals toward sexual forms of jealousy. For anyone with lower gynephilic orientation, any perceptible levels of androphilic orientation, or female sex, the general response to infidelity focuses on emotional and relational aspects rather than the sexual aspect. However, these shifts are not completely dichotomous or binary. A certain level of sexual jealousy, on average, remains stable across all other profiles of sexual orientation and sex, which also needs to be addressed in the future.

Four different indicators (psychological dials) of feminization-masculinization were linked to both continuous conceptions of sexual orientation and jealousy responses for men, with more femininity appearing alongside non-heterosexuality and emotional expression of jealousy. In strong contrast, psychological feminization was only linked to sexual orientation among women. Being more masculine may not lead to more sexual jealousy when that masculinity exists within a woman.

Various individual differences in aspects of psychological development and function related to sex have an impact on established sex differences. The current findings reinforce the concept that female versus male sexual psychology is not merely dimorphic. Rather, sex and gender are the results of many different dials that may interact to a large degree, but are partially modular or independent of each other, providing a rich and varied expression of sexual psychology (Schmitt, 2017).

## Supplementary Information

Below is the link to the electronic supplementary material.

Supplementary file1 (DOCX 13 kb)

## Data Availability

Data uploaded to data repository at https://osf.io/8uarp/
