# Supplementary material for: Male Sex, Masculinization, Sexual Orientation, and Gynephilia Synergistically Predict Increased Sexual Jealousy
Source: Arch Sex Behav. 2025 Aug 22;54(8):3189–203. doi: 10.1007/s10508-025-03225-z (PMC12484348; doi:10.1007/s10508-025-03225-z)
Supplement: Supplementary file 1 — Supplementary file1 (DOCX 13 kb) [file 10508_2025_3225_MOESM1_ESM.docx]

**Supplementary Material**

*Means (and SDs) for Men and Women across Self-Identified Sexual Orientation Group*

|  |  | Heterosexual | | Bisexual | | Gay/Lesbian | | Pansexual | |
| --- | --- | --- | --- | --- | --- | --- | --- | --- | --- |
| Variable |  | Men  n = 1555 | Women  n = 1741 | Men  n = 107 | Women  n = 279 | Men  n = 430 | Women  n = 216 | Men  n = 33 | Women  n = 104 |
| Age (16–80)  Currently partnered (%)  S-E (± 20)  Nonconformity (1–7)  M-F Occ Pref (1–7)  Perceived M-F (1–7)  Gyne-Androphilia (± 3) |  | 36.08  (13.62)  64.1  3.80  (4.86)  2.19  (0.89)  4.46  (0.74)  5.35  (1.06)  2.69  (0.44) | 37.41  (13.39)  70.6  –3.14  (5.04)  3.13  (1.30)  3.35  (0.75)  2.71  (1.03)  –2.32  (0.62) | 33.79  (13.49)  48.1  1.71  (5.20)  2.74  (1.03)  4.08  (0.77)  5.09  (1.03)  0.53  (1.35) | 29.83  (9.68)  65.1  –2.03  (5.12)  3.59  (1.36)  3.47  (0.80)  3.13  (1.11)  –0.30  (1.03) | 33.77  (11.54)  48.3  0.75  (5.20)  3.58  (1.30)  3.78  (0.79)  4.69  (1.23)  –2.64  (0.45) | 30.42  (9.54)  56.9  –2.74  (5.15)  4.00  (1.55)  3.74  (0.84)  3.76  (1.34)  2.48  (0.47) | 30.91  (10.45)  60.6  3.61  (5.54)  3.24  (1.15)  3.77  (0.73)  4.68  (1.01)  0.88  (1.43) | 27.18  (7.39)  59.5  –1.90  (5.62)  3.81  (1.16)  3.41  (0.77)  3.43  (1.21)  –0.07  (1.12) |

*Note*. S-E = Systemizing-Empathizing, M-F Occ Pref = Male-Female Occupation Preferences, Perceived M-F = Perceived Masculine-Feminine. Scale score range in parenthesis.
